# Supplementary material for: Cryo EM Analysis Reveals Inherent Flexibility of Authentic Murine Papillomavirus Capsids
Source: Viruses. 2021 Oct 7;13(10):2023. doi: 10.3390/v13102023 (PMC8537154; doi:10.3390/v13102023)
Supplement: Supplementary file 1 [file viruses-13-02023-s001.zip › viruses-1367909-supplementary.pdf]

Supplementary Information

Supplementary Table S1. Cryo-EM data collection, refinement and validation statistics.

**Data Collection and Processing**	**Icosahedral**	**Pentavalent Capsomer**	**Hexavalent Capsomer**
Magnification	59,000	59,000	59,000
Voltage (kV)	300	300	300
Electron Exposure (e-/Å^2)	45	45	45
Defocus Range (um)	0.5-3.0	0.5-3.0	0.5-3.0
Pixel Size (Å)	1.1	1.1	1.1
Symmetry Imposed	I1	C5	C1
Initial Particle Number	16,978	-	-
Final Particle Number	10,181	-	-
Sub-particles per Particle	-	12	60
Final Sub-particle Number	-	122,172	610,860
Map Resolution	4.41	3.4	3.3
FSC Threshold	0.143	0.143	0.143

**Refinement**	**Recombined Capsid**
Model composition	
Non-hydrogen atoms	22959
Protein Residues	2867
B-Factors	
Protein	53.4
Validation	
MolProbity Score	1.71
Clash Score	6.65
Rotamer Outliers (%)	0.04
Ramachandran Plot	
Favored (%)	95.10
Outliers (%)	0.28

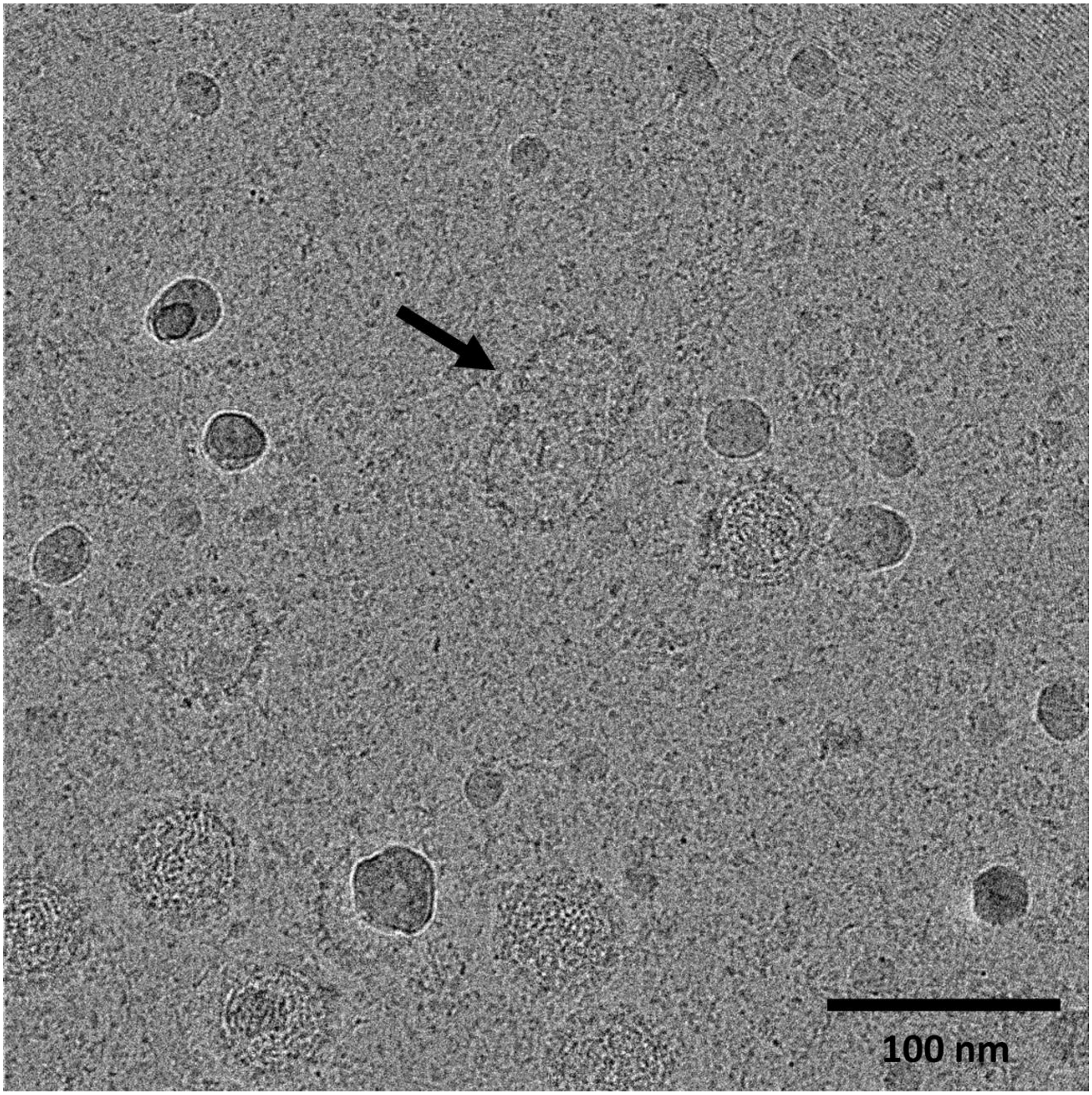

Supplementary Figure S1. Micrograph with rod like structure. Black arrow points to a rod like structure that is seen within the dataset.

A:F

Level	Count	Std Dev	MeanAbsDif to Mean	MeanAbsDif to Median
HPV16 Quasivirus	43679	0.0407412	0.0289734	0.0289458
MmuPV1 Native Virus	2681	0.0254883	0.0164075	0.0163573

Test	F Ratio	DFNum	DFDen	p-Value
O'Brien[.5]	233.1095	1	46358	<.0001*
Brown-Forsythe	500.3583	1	46358	<.0001*
Levene	501.7482	1	46358	<.0001*
Bartlett	853.3847	1	.	<.0001*
F Test 2-sided	2.5550	43678	2680	<.0001*

B:Null

Level	Count	Std Dev	MeanAbsDif to Mean	MeanAbsDif to Median
HPV16 Quasivirus	87358	0.0358045	0.0255266	0.0255079
MmuPV1 Native Virus	5108	0.0230426	0.0151967	0.0151844

Test	F Ratio	DFNum	DFDen	p-Value
O'Brien[.5]	401.7804	1	92464	<.0001*
Brown-Forsythe	835.8814	1	92464	<.0001*
Levene	841.2870	1	92464	<.0001*
Bartlett	1460.2527	1	.	<.0001*
F Test 2-sided	2.4144	87357	5107	<.0001*

C:D

Level	Count	Std Dev	MeanAbsDif to Mean	MeanAbsDif to Median
HPV16 Quasivirus	87358	0.0375155	0.0262676	0.0262637
MmuPV1 Native Virus	4825	0.0254530	0.0157794	0.0157787

Test	F Ratio	DFNum	DFDen	p-Value
O'Brien[.5]	304.5668	1	92181	<.0001*
Brown-Forsythe	716.1693	1	92181	<.0001*
Levene	717.7739	1	92181	<.0001*
Bartlett	1101.6752	1	.	<.0001*
F Test 2-sided	2.1724	87357	4824	<.0001*

E:E

Level	Count	Std Dev	MeanAbsDif to Mean	MeanAbsDif to Median
HPV16 Quasivirus	43679	0.0392467	0.0282713	0.0282526
MmuPV1 Native Virus	2765	0.0274450	0.0187063	0.0186933

Test	F Ratio	DFNum	DFDen	p-Value
O'Brien[.5]	181.0447	1	46442	<.0001*
Brown-Forsythe	328.5869	1	46442	<.0001*
Levene	330.0078	1	46442	<.0001*
Bartlett	542.9211	1	.	<.0001*
F Test 2-sided	2.0449	43678	2764	<.0001*

Diameter

Level	Count	Std Dev	MeanAbsDif to Mean	MeanAbsDif to Median
HPV16 Quasivirus	50224	0.0090671	0.0072330	0.0072330
MmuPV1 Native Virus	3967	0.0106710	0.0078790	0.0078165

Test	F Ratio	DFNum	DFDen	p-Value
O'Brien[.5]	181.3232	1	54189	<.0001*
Brown-Forsythe	39.5270	1	54189	<.0001*
Levene	48.7183	1	54189	<.0001*
Bartlett	214.1058	1	.	<.0001*
F Test 2-sided	1.3851	3966	50223	<.0001*

Supplementary Figure S2. Statistical Tests of Unequal Variances. Comparison of HPV16 QV and MmuPV1 for intercapsomere distances and diameter compared through unequal variances.

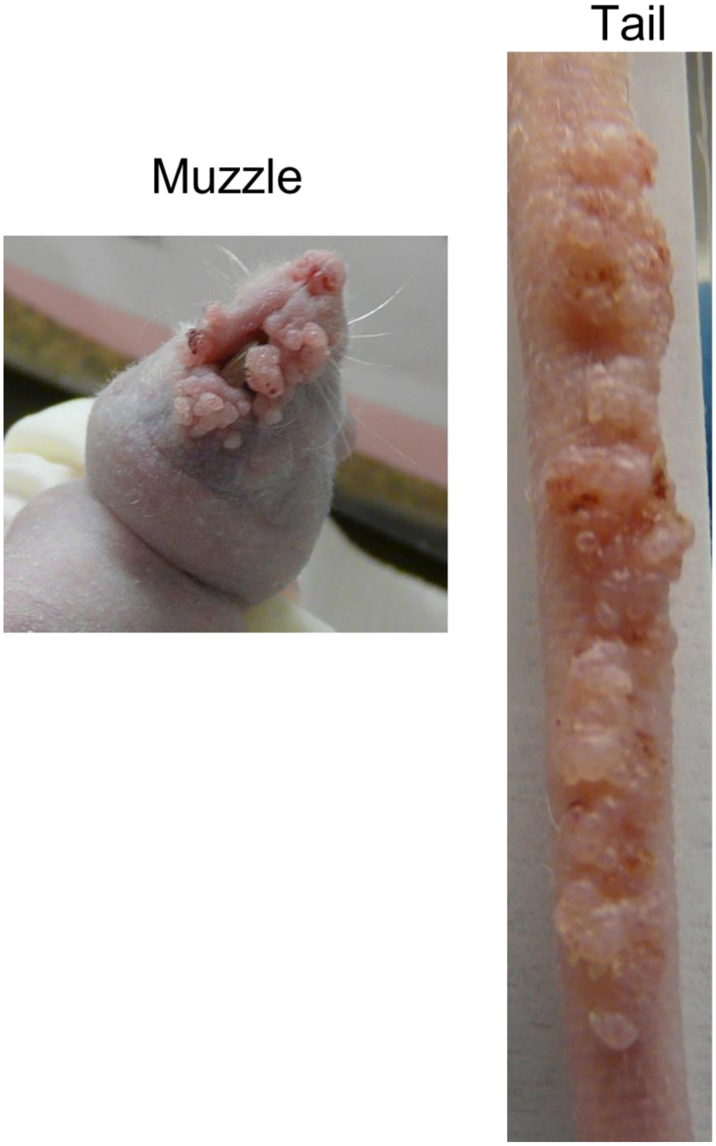

Supplementary Figure S3. Lesions virus sample was purified from. Virus sample was purified from mouse wart homogenate from the muzzle and tail of infected mice.

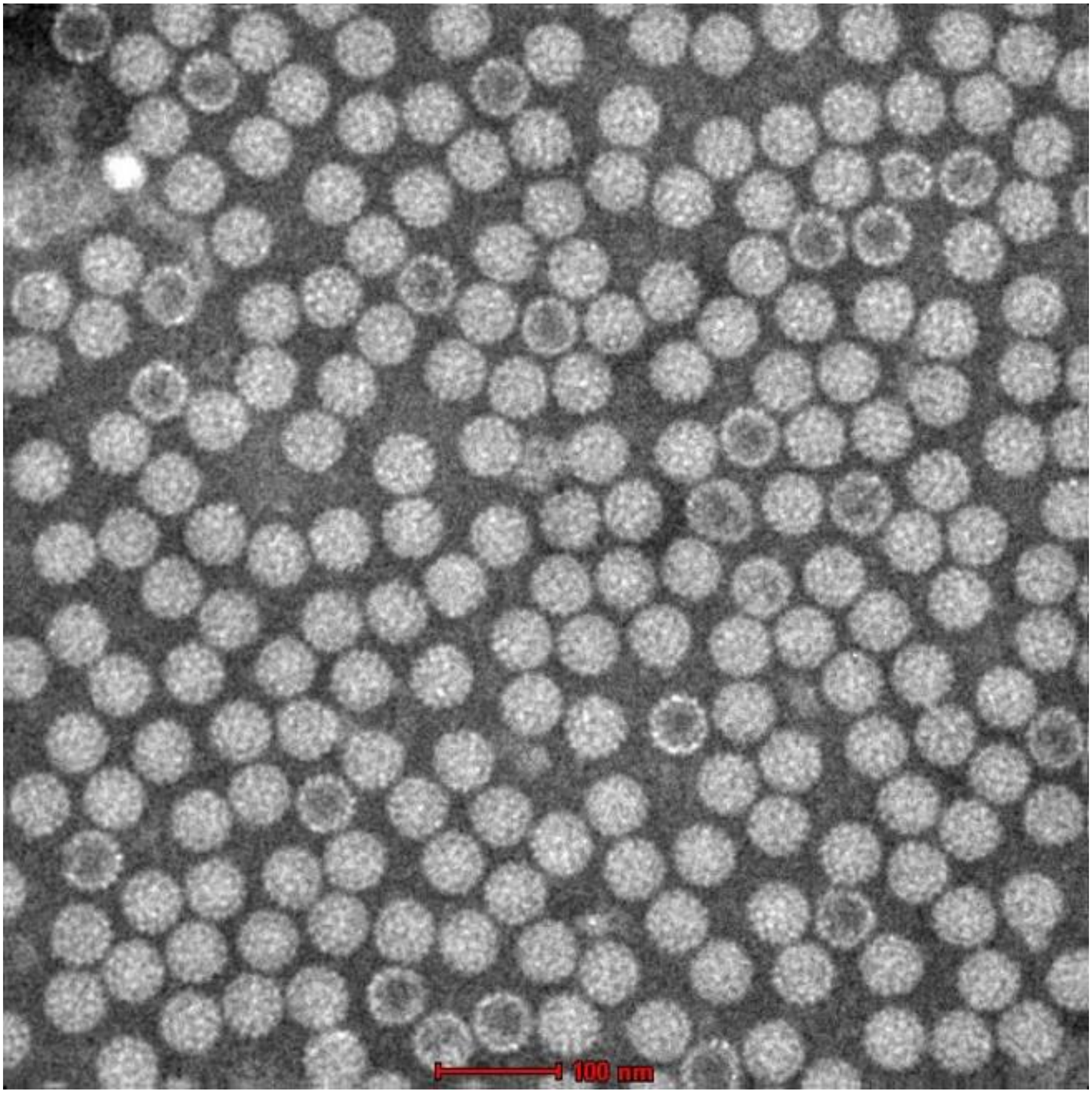

Supplementary Figure S4. TEM negative stain image of virus before vitrification.

```
Sequence Alignment
AYV61481.1  L1 [Human papillomavirus type 16]
ATU83344.1  viral protein L1 [Mus musculus papillomavirus type 1]

AYV61481.1    1 MQVTFIYILVITCYENDVNVYHIFFQMSLWLPSEATVYLPP-VPVSKVVSTDEYVARTNI
ATU83344.1    1 --------------------------MAMWTPQTGKLYLPPTTPVAKVQSTDEYVYPTSL

AYV61481.1   60 YYHAGTSRLLAVGHPYFPIKKPNNNKILVPKVSGLQYRVFRIHLPDPNKFGFPDTSFYNP
ATU83344.1   35 FCHAHTDRLLTVGHPFFSVI--DNDKVTVPKVSGNQYRVFRLKFPDPNKFALPQKDFYDP

AYV61481.1  120 DTQRLVWACVGVEVGRGQPLGVGISGHPLLNKLDDTENASAYAANAGVDNRECISMDYKQ
ATU83344.1   93 EKERLVWRLRGLEIGRGGPLGIGTTGHPLFNKLGDTENPNKYQQG-SKDNRQNTSMDPKQ

AYV61481.1  180 TQLCLIGCKPPIGEHWGKGSPCTNVAVNPGDCPPLELINTVIQDGDMVDTGFGAMDFTTL
ATU83344.1  152 TQLFIVGCEPPTGEHWDVAKPCG--ALEKGDCPPIQLVNSVIEDGDMCDIGFGNMNFKEL

AYV61481.1  240 QANKSEVPLDICTSICKYPDYIKMVSEPYGDSLFFYLRREQMFVRHLFNRAGAVGENVPD
ATU83344.1  210 QQDRSGVPLDIVSTRCKWPDFLKMTNEAYGDKMFFFGRREQVYARHFFTRNGSVGEPIPN

AYV61481.1  300 D-----LYIKG--SGSTANLASSNYFPTPSGSMVTSDAQIFNKPYWLQRAQGHNNGICWG
ATU83344.1  270 SVSPSDFYYAPDSTQDQKTLAPSVYFGTPSGSLVSSDGQLFNRPFWLQRAQGNNNGVCWH

AYV61481.1  353 NQLFVTVVDTTRSTNMSLCAAISTSE-TTYKNTNFKEYLRHGEEYDLQFIFQLCKITLTA
ATU83344.1  330 NELFVTVVDNTRNTNFTISQQTNTPNPDTYDSTNFKNYLRHVEQFELSLIAQLCKVPLDP

AYV61481.1  412 DVMTYIHSMNSTILEDWNFGLQPPPGGTLEDTYRFVTSQAIACQKHTPPAPKEDPLKKYT
ATU83344.1  390 GVLAHINTMNPTILENWNLGFVPPPQQSISDDYRYITSSATRCPDQNPPKEREDPYKGLI

AYV61481.1  472 FWEVNLKEKFSADLDQFPLGRKFLLQAGLKAKPKFTLGKRKATPTTSSTSTTAKRKKRKL
ATU83344.1  450 FWEVDLTERFSQDLDQFALGRKFLYQAGIRTAVTGRGVKRAASTTSASSRRVVKRKRGSK
```

Supplementary Figure S5. Sequence alignment of HPV16 and MmuPV1 L1, major capsid protein.
